# Supplementary material for: Exploring the Role of Physical Activity in Mediating the Association between Educational Level and Health-Related Quality of Life in an Adult Lifespan Sample from Madeira Island
Source: Int J Environ Res Public Health. 2022 Jun 22;19(13):7608. doi: 10.3390/ijerph19137608 (PMC9265542; doi:10.3390/ijerph19137608)
Supplement: Supplementary file 1 [file ijerph-19-07608-s001.zip › ijerph-1752298-supplementary.pdf]

Supplementary material

Tables

Table S1. Relationships between educational attainment (coded in 3 categories) and PA in health-related quality of life (N = 364).

**Pathways Model 1 key**	**Indirect effect**	**PM or PS**
EDUC -> Total PA -> (outcome PCS)	-.0972 (95% CI -.1985, -.0203)	PS = -8.4%
EDUC -> Total PA -> (outcome MCS)	-.1074 (95% CI -.2330, .-.0141)	PS = -45.9%
EDUC -> Total PA -> (outcome Total SF 12 score)	-.2046 (95% CI -.4243, -.0425)	PS = -14.8%
**Pathways Model 2 key**		
**Outcome PCS**		
Total (model)	.1358 (95% CI -.0583, .3307)	
EDUC->PA at work->PCS	-.0035 (95% CI -.1396, .1113)	
EDUC->Sports PA->PCS	.1796 (95% CI .0725, .3273)	PM = 15.6%
EDUC->Leisure-time PA->PCS	.0049 (95% CI-.0523, .0657)	
EDUC->PA at work->Sports PA->PCS	-.0445 (95% CI -.0922, -.0074)	PS = -3.9%
EDUC->PA at work->Leisure-time PA->PCS	.0010 (95% CI -.0136, .0137)	
EDUC->Sports PA->Leisure-time PA->PCS	-.0024 (95% CI -.0305, .0231)	
EDUC->PA at work->Sports PA->Leisure-time PA->PCS	.0006 (95% CI -.0063, .0079)	
**Outcome MCS**		
Total (model)	.0406 (95% CI -.2543, .3253)	
EDUC ->PA at work->MCS	-.0024 (95% CI -.2129, .1944)	
EDUC->Sports PA->MCS	.1174 (95% CI -.0023, .2723)	PM = 50.2%
EDUC->Leisure-time PA->MCS	-.0533 (95% CI -.1576, .0214)	
EDUC->PA at work->Sports PA->MCS	-.0291 (95% CI -.0749, .0006)	
EDUC->PA at work->Leisure-time PA->MCS	-.0113 (95% CI -.0411, .0053)	
EDUC->Sports PA>Leisure-time PA->MCS	.0256 (95% CI -.0094, .0807)	
EDUC->PA at work->Sports PA->Leisure-time PA->MCS	-.0063 (95% CI -.0215, .0024)	
**Outcome SF-12 score**		
Total (model)	.1764 (95% CI -.2102, .6083)	
EDUC->PA at work->SF-12 score	-.0059 (95% CI -.2907, .2956)	
EDUC->Sports PA->SF-12 score	.2970 (95% CI .1058, .5751)	PM = 21.4%
EDUC->Leisure-time PA->SF-12 score	.0484 (95% CI-.1670, .0719)	
EDUC->PA at work->Sports PA->SF-12 score	-.0736 (95% CI -.1730, -.0099)	PS = -5.3%
EDUC->PA at work->Leisure-time PA->SF-12 score	.0103 (95% CI -.0473, .0137)	
EDUC->Sports PA->Leisure-time PA->SF-12 score	-.0232 (95% CI -.0309, .0881)	
EDUC->PA at work->Sports PA->Leisure-time PA->SF-12 score	.0058 (95% CI -.0253, .0082)	

We show unstandardized regression coefficients (b) and bootstrap confidence intervals for the association. Education is codified into three levels: (1) < 3rd cycle of education, (2) secondary education, (3) higher education. PA is physical activity. PCS and MCS are Physical and Mental Component Summaries of SF-12. SF-12 score corresponds to the total score on the SF-12 scale. Proportion mediated (PM) or Proportion Suppressed (PS) represent the percentage of the total effect of Educational attainment on health-related quality of life by each pathway.

Table S2. Relationships between educational attainment and PA at work and total leisure-time PA (model 3) in health-related quality of life (N = 364).

Pathways coding education in 8 categories	Indirect effect	PM or PS
**Outcome PCS**		
Total (model)	-.0048 (95% CI -.0870, .0740)	
EDUC->PA at work->PCS	-.0020 (95% CI -.0692, .0652)	
EDUC->Total leisure-time PA->PCS	.0189 (95% CI -.0314, .0735)	
EDUC->PA at work->Total leisure-time PA->PCS	-.0217 (95% CI -.0445, -.0045)	PS = -3.4%
**Outcome MCS**		
Total (model)	.0096 (95% CI -.1209, .1135)	
EDUC->PA at work->MCS	-.0066 (95% CI -.1149, .1042)	
EDUC->Total leisure-time PA->MCS	.0202 (95% CI -.0337, .0912)	
EDUC->PA at work->Total leisure-time PA->MCS	-.0232 (95% CI -.0519, -.0043)	PS = -13.4%
**Outcome PCS**		
Total (model)	-.0144 (95% CI -.1909, .1720)	
EDUC->PA at work->SF-12 score	-.0087 (95% CI -.1587, .1472)	
EDUC->Total leisure-time PA->SF-12 score	.0391 (95% CI -.0595, .1530)	
EDUC->PA at work->Total leisure-time PA->SF-12 score	-.0448 (95% CI -.0898, -.0100)	PS = -5.5%
**Pathways coding education in 3 categories**		
**Outcome PCS**	.0107 (95% CI -.1564, .1552)	
Total (model)	-.0045 (95% CI -.1499, .1288)	
EDUC->PA at work->PCS	.0570 (95% CI -.0326, .1487)	
EDUC->Total leisure-time PA->PCS	-.0418 (95% CI -.0850, -.0102)	PS = -3.6%
EDUC->PA at work->Total leisure-time PA->PCS		
**Outcome MCS**		
Total (model)	.0143 (95% CI -.2098, .2379)	
EDUC->PA at work->MCS	-.0026 (95% CI -.2161, .2037)	
EDUC->Total leisure-time PA->MCS	.0635 (95% CI -.0238, .1842)	
EDUC->PA at work->Total leisure-time PA->MCS	-.0446 (95% CI -.1023, -.0081)	PS = -19.1%
**Outcome PCS**		
Total (model)	.0250 (95% CI -.3531, .3885)	
EDUC->PA at work->SF-12 score	-.0071 (95% CI -.3159, .3202)	
EDUC->Total leisure-time PA->SF-12 score	.1205 (95% CI -.0506, .3510)	
EDUC->PA at work->Total leisure-time PA->SF-12 score	-.0884 (95% CI -.1802, -.0209)	PS = -6.4%

We show unstandardized regression coefficients (b) and bootstrap confidence intervals for the association. Education codified into three levels corresponds to: (1) < 3rd cycle of education, (2) secondary education, (3) higher education. PA is physical activity. PCS and MCS are Physical and Mental Component Summaries of SF-12. SF-12 score corresponds to the total score on the SF-12 scale. Proportion mediated (PM) or Proportion Suppressed (PS) represent the percentage of the total effect of Educational attainment on health-related quality of life by each pathway.

Table S3. Total and direct effects of educational attainment on health-related quality of life in the Madeiran population by age group.

**Madeiran population aged between 18 and 44 years old (N = 185)**		
	**Coding education in 8 categories**	**Coding education in 3 categories**
**Total effect**		
EDUC -> (outcome PCS)	.5538 (95% CI .2331, .8745) ***	.9295 (95% CI .4473, 1.4117) ***
EDUC -> (outcome MCS)	.1367 (95% CI -.3413, .6148)	.0669 (95% CI -.6578, .7916)
EDUC -> (outcome SF-12 score)	.6905 (95% CI -.0056, 1.3897)	.9964 (95% CI -.0591, 2.0519)
**Direct effect**		
**Model 1**		
EDUC -> (outcome PCS)	.6368 (95% CI .3102, .9634) ***	1.0286 (95% CI .5416, 1.5157) ***
EDUC -> (outcome MCS)	.2535 (95% CI -.2340, .7411)	.2038 (95% CI -.5296, .9372)
EDUC -> (outcome SF-12 score)	.8904 (95% CI .1835, 1.5672) *	1.2324 (95% CI .1686, 2.2962) *
**Model 2**		
EDUC -> (outcome PCS)	.4138 (95% CI .0796, .7480) *	.6142 (95% CI .1041, 1.1243) *
EDUC -> (outcome MCS)	.0779 (95% CI -.4474, .6032)	-.1759 (95% CI -.9767, .6248)
EDUC -> (outcome SF-12 score)	.4917 (95% CI -.2497, 1.2331)	.4382 (95% CI -.6959, 1.5724)
**Model 3**		
EDUC -> (outcome PCS)	.5449 (95% CI .1949, .8949) **	.9020 (95% CI .3811, 1.4230) ***
EDUC -> (outcome MCS)	.1740 (95% CI -.3504, .6984)	.0596 (95% CI -.7266, .8457)
EDUC -> (outcome SF-12 score)	.7189 (95% CI -.0396, 1.4774)	.9616 (95% CI -.1765, 2.0997)
**Madeiran population ≥ 45 years old (N = 179)**		
**Total effect**		
EDUC -> (outcome PCS)	.6171 (95% CI .3114, .9197) ***	1.2165 (95% CI .5652, 1.8678) ***
EDUC -> (outcome MCS)	.0536 (95% CI -.3519, .4591)	.0574 (95% CI -.8092, .9240)
EDUC -> (outcome SF-12 score)	.6706 (95% CI .0351, 1.3062) *	1.2739 (95% CI -.0879, 2.6357)
**Direct effect**		
**Model 1**		
EDUC -> (outcome PCS)	.6809 (95% CI .3799, .9820) ***	1.3228 (95% CI .6758, 1.9699) ***
EDUC -> (outcome MCS)	.1095 (95% CI -.2987, .5176)	.1546 (95% CI -.7147, 1.0238)
EDUC -> (outcome SF-12 score)	.7904 (95% CI .1555, 1.4252) *	1.4774 (95% CI .1205, 2.8344)
**Model 2**		
EDUC -> (outcome PCS)	.6031 (95% CI .2894, .9167) ***	1.1352 (95% CI .4617, 1.8088) **
EDUC -> (outcome MCS)	.0899 (95% CI -.3348, .5145)	.0701 (95% CI -.8337, .9740)
EDUC -> (outcome SF-12 score)	.6929 (95% CI .0322, 1.3537) *	1.2054 (95% CI -.2063, 2.6171)
**Model 3**		
EDUC -> (outcome PCS)	.6097 (95% CI .3020, .9175) ***	1.564 (95% CI .5652, 1.8678) **
EDUC -> (outcome MCS)	.0352 (95% CI -.3838, .4542)	-.0219 (95% CI -.9184, .8746)
EDUC -> (outcome SF-12 score)	.6449 (95% CI -.0045, 1.2943)	1.1345 (95% CI -.2598, 2.5288)

We show unstandardized regression coefficients (b) and bootstrap confidence intervals for the association. Education codified into three levels corresponds to: (1) < 3rd cycle of education, (2) secondary education, (3) higher education. PCS and MCS are Physical and Mental Component Summaries of SF-12. SF-12 score corresponds to the total score on the SF-12 scale. *P < .05, **P ≤ .01, *** P < .001.

Table S4. Relationships between educational attainment and PA in health-related quality of life in the Madeiran population aged between 18 and 44 years old (N = 185).

Pathways Model 1 key	Indirect effect	PM or PS
EDUC -> Total PA -> (outcome PCS)	-.0830 (95% CI -.1893, -.0053)	PS = -14.10%
EDUC -> Total PA -> (outcome MCS)	-.1168 (95% CI -.2741, -.0085)	PS = -85.44%
EDUC -> Total PA -> (outcome SF-12 score)	-.1998 (95% CI -.4376, -.0330	PS = -28.94%
**Pathways Model 2 key**		
**Outcome PCS**		
Total (model)	.1400 (95% CI -.0517, .3219)	
EDUC->PA at work->PCS	.0205 (95% CI -.1421, .1634)	
EDUC->Sports PA->PCS	.1282 (95% CI -.0110, .2804)	
EDUC->Leisure-time PA->PCS	.0455 (95% CI -.0180, .1382)	
EDUC->PA at work->Sports PA->PCS	-.0522 (95% CI -.1294, .0153	
EDUC->PA at work->Leisure-time PA->PCS	.0104 (95% CI -.0152, .0420	
EDUC->Sports PA->Leisure-time PA->PCS	-.0209 (95% CI -.0582, .0022	
EDUC->PA at work->Sports PA->Leisure-time PA->PCS	.0085 (95% CI -.0021, .0278	
**Outcome MCS**		
Total (model)	.0589 (95% CI -.2726, .4058)	
EDUC ->PA at work->MCS	-.0264 (95% CI -.3153, .2587)	
EDUC->Sports PA->MCS	.1120 (95% CI -.0099, .3042)	
EDUC->Leisure-time PA->MCS	.0197 (95% CI -.0444, .1313)	
EDUC->PA at work->Sports PA->MCS	-.0456 (95% CI -.1420, .0084)	
EDUC->PA at work->Leisure-time PA->MCS	.0045 (95% CI -.0159, .0375)	
EDUC->Sports PA>Leisure-time PA->MCS	-.0091 (95% CI -.0542, .0214)	
EDUC->PA at work->Sports PA->Leisure-time PA->MCS	.0037 (95% CI -.0097, .0234)	
**Outcome SF-12 score**		
Total (model)	.1988 (95% CI -.2980, .7054)	
EDUC->PA at work->SF-12 score	-.0060 (95% CI -.4120, .4092)	
EDUC->Sports PA->SF-12 score	.2402 (95% CI -.0278, .5798)	
EDUC->Leisure-time PA->SF-12 score	.0652 (95% CI -.0285, .2556)	
EDUC->PA at work->Sports PA->SF-12 score	-.0978 (95% CI -.2695, .0147)	
EDUC->PA at work->Leisure-time PA->SF-12 score	.0150 (95% CI -.0264, .0736)	
EDUC->Sports PA->Leisure-time PA->SF-12 score	-.0300 (95% CI -.1057, .0085)	
EDUC->PA at work->Sports PA->Leisure-time PA->SF-12 score	.0122 (95% CI -.0037, .0466)	

We show unstandardized regression coefficients (b) and bootstrap confidence intervals for the association. PA is physical activity. PCS and MCS are Physical and Mental Component Summaries of SF-12. SF-12 score corresponds to the total score on the SF-12 scale. Proportion mediated (PM) or Proportion Suppressed (PS) represent the percentage of the total effect of Educational attainment on health-related quality of life by each pathway.

Table S5. Relationships between educational attainment (coded in 3 categories) and PA in health-related quality of life in the Madeiran population aged between 18 and 44 years old (N = 185).

Pathways Model 1 key	Indirect effect	PM or PS
EDUC -> Total PA -> (outcome PCS)	-,0991 (95% CI -.2402, -.0026)	PS = -10.7%
EDUC -> Total PA -> (outcome MCS)	-.1368 (95% CI -.3600, .0054)	
EDUC -> Total PA -> (outcome SF-12 score)	-.2360 (95% CI -.5718, -.0009)	PS = -23.7%
**Pathways Model 2 key**		
**Outcome PCS**		
Total (model)	.3153 (95% CI .0175, .6283)	PM = 33.9%
EDUC->PA at work->PCS	.0379 (95% CI -.1986, .2371)	
EDUC->Sports PA->PCS	.2892 (95% CI .0730, .5335)	PM = 31.1%
EDUC->Leisure-time PA->PCS	.0988 (95% CI -.0012, .2717)	
EDUC->PA at work->Sports PA->PCS	-.0852 (95% CI -.2015, .0022)	
EDUC->PA at work->Leisure-time PA->PCS	.0092 (95% CI -.0302, .0509)	
EDUC->Sports PA->Leisure-time PA->PCS	-.0489 (95% CI -.1323, -.0005)	PS = -5.3%
EDUC->PA at work->Sports PA->Leisure-time PA->PCS	.0144 (95% CI -.0013, .0457)	
**Outcome MCS**		
Total (model)	.2429 (95% CI -.3119, .8094)	
EDUC ->PA at work->MCS	.0097 (95% CI -.4116, .4101)	
EDUC->Sports PA->MCS	.2759 (95% CI .0384, .5901)	PM = 412.4%
EDUC->Leisure-time PA->MCS	.0519 (95% CI -.0742, .2669)	
EDUC->PA at work->Sports PA->MCS	-.0813 (95% CI -.2270, .0049)	
EDUC->PA at work->Leisure-time PA->MCS	.0048 (95% CI -.0282, .0510)	
EDUC->Sports PA>Leisure-time PA->MCS	-.0257 (95% CI -.1202, .0415)	
EDUC->PA at work->Sports PA->Leisure-time PA->MCS	.0076 (95% CI -.0152, .0439)	
**Outcome SF-12 score**		
Total (model)	.5582 (95% CI -.2082, 1.3777)	
EDUC->PA at work->SF-12 score	.0476 (95% CI -.5236, .5691)	
EDUC->Sports PA->SF-12 score	.5651 (95% CI .1611, 1.0813)	PM = 56.7%
EDUC->Leisure-time PA->SF-12 score	.1508 (95% CI -.0237, .4731)	
EDUC->PA at work->Sports PA->SF-12 score	-.1666 (95% CI -.4376, -.0009)	PS = -16.7%
EDUC->PA at work->Leisure-time PA->SF-12 score	.0140 (95% CI -.0498, .0844)	
EDUC->Sports PA->Leisure-time PA->SF-12 score	-.0746 (95% CI -.2248, .0161)	
EDUC->PA at work->Sports PA->Leisure-time PA->SF-12 score	.0220 (95% CI -.0060, .0774)	

We show unstandardized regression coefficients (b) and bootstrap confidence intervals for the association. Education is codified into three levels: (1) < 3rd cycle of education, (2) secondary education, (3) higher education. PA is physical activity. PCS and MCS are Physical and Mental Component Summaries of SF-12. SF-12 score corresponds to the total score on the SF-12 scale. Proportion mediated (PM) or Proportion Suppressed (PS) represent the percentage of the total effect of Educational attainment on health-related quality of life by each pathway.

Table S6. Relationships between educational attainment and PA in health-related quality of life in the Madeiran population ≥ 45 years old (N = 179).

**Pathways Model 1 key**	**Indirect effect**	**PM or PS**
EDUC -> Total PA -> (outcome PCS)	-.0639 (95% CI -.1636, -.0033)	PS = -10.4%
EDUC -> Total PA -> (outcome MCS)	-.0559 (95% CI -.1706, .0095)	
EDUC -> Total PA -> (outcome SF-12 score)	-.1197 (95% CI -.3139, .0042)	
**Pathways Model 2 key**		
**Outcome PCS**		
Total (model)	.0140 (95% CI -.1325, .1638)	
EDUC->PA at work->PCS	.0160 (95% CI -.0928, .1236)	
EDUC->Sports PA->PCS	.0457 (95% CI -.0061, .1428)	
EDUC->Leisure-time PA->PCS	-.0350 (95% CI -.1188, .0100)	
EDUC->PA at work->Sports PA->PCS	-.0099 (95% CI -.0395, .0058)	
EDUC->PA at work->Leisure-time PA->PCS	-.0120 (95% CI -.0363, .0018)	
EDUC->Sports PA->Leisure-time PA->PCS	.0118 (95% CI -.0016, .0349)	
EDUC->PA at work->Sports PA->Leisure-time PA->PCS	-.0026 (95% CI -.0115, .0013)	
**Outcome MCS**		
Total (model)	-.0363 (95% CI -.2272, .1374)	
EDUC ->PA at work->MCS	.0333 (95% CI -.1184, .1671)	
EDUC->Sports PA->MCS	.0056 (95% CI -.0677, .0931)	
EDUC->Leisure-time PA->MCS	-.0685 (95% CI -.1922, .0116)	
EDUC->PA at work->Sports PA->MCS	-.0012 (95% CI -.0252, .0153)	
EDUC->PA at work->Leisure-time PA->MCS	-.0235 (95% CI -.0573, .0020)	
EDUC->Sports PA>Leisure-time PA->MCS	.0230 (95% CI -.0047, .0776)	
EDUC->PA at work->Sports PA->Leisure-time PA->MCS	-.0050 (95% CI -.0189, .0025)	
**Outcome SF-12 score**		
Total (model)	-.0223 (95% CI -.3560, .2698)	
EDUC->PA at work->SF-12 score	.0493 (95% CI -.1822, .2694)	
EDUC->Sports PA->SF-12 score	.0513 (95% CI -.0450, .2289)	
EDUC->Leisure-time PA->SF-12 score	-.1035 (95% CI -.2941, .0130)	
EDUC->PA at work->Sports PA->SF-12 score	-.0112 (95% CI -.0635, .0120)	
EDUC->PA at work->Leisure-time PA->SF-12 score	-.0354 (95% CI -.0922, .0038)	
EDUC->Sports PA->Leisure-time PA->SF-12 score	.0348 (95% CI -.0083, .1069)	
EDUC->PA at work->Sports PA->Leisure-time PA->SF-12 score	-.0076 (95% CI -.0256, .0040)	

We show unstandardized regression coefficients (b) and bootstrap confidence intervals for the association.. PA is physical activity. PCS and MCS are Physical and Mental Component Summaries of SF-12. SF-12 score corresponds to the total score on the SF-12 scale. Proportion mediated (PM) or Proportion Suppressed (PS) represent the percentage of the total effect of Educational attainment on health-related quality of life by each pathway.

Table S7. Relationships between educational attainment (coded in 3 categories) and PA in health-related quality of life in the Madeiran population ≥ 45 years old (N = 179).

**Pathways Model 1 key**	**Indirect effect**	**PM or PS**
EDUC -> Total PA -> (outcome PCS)	-.1064 (95% CI -.2948, .0049)	
EDUC -> Total PA -> (outcome MCS)	-.0972 (95% CI -.3088, .0341)	
EDUC -> Total PA -> (outcome SF-12 score)	-.2035 (95% CI -.5806, .0359)	
**Pathways Model 2 key**		
**Outcome PCS**		
Total (model)	.0812 (95% CI -.2346, .3918)	
EDUC->PA at work->PCS	.0428 (95% CI -.1859, .2712)	
EDUC->Sports PA->PCS	.1085 (95% CI -.0197, .3099)	
EDUC->Leisure-time PA->PCS	-.0416 (95% CI -.1930, .0351)	
EDUC->PA at work->Sports PA->PCS	-.0225 (95% CI -.0854, .0109)	
EDUC->PA at work->Leisure-time PA->PCS	-.0242 (95% CI -.0757, .0048)	
EDUC->Sports PA->Leisure-time PA->PCS	.0230 (95% CI -.0039, .0747)	
EDUC->PA at work->Sports PA->Leisure-time PA->PCS	-.0048 (95% CI -.0202, .0029)	
**Outcome MCS**		
Total (model)	-.0127 (95% CI -.4283, .3787)	
EDUC ->PA at work->MCS	.0796 (95% CI -.2251, .3865)	
EDUC->Sports PA->MCS	.0156 (95% CI -.1441, .1989)	
EDUC->Leisure-time PA->MCS	-.0915 (95% CI -.3382, .0665)	
EDUC->PA at work->Sports PA->MCS	-.0032 (95% CI -.0483, .0303)	
EDUC->PA at work->Leisure-time PA->MCS	-.0533 (95% CI -.1297, .0028)	
EDUC->Sports PA>Leisure-time PA->MCS	.0506 (95% CI -.0073, .1464)	
EDUC->PA at work->Sports PA->Leisure-time PA->MCS	-.0105 (95% CI -.0433, .0055)	
**Outcome SF-12 score**		
Total (model)	.0685 (95% CI -.5645, .6476)	
EDUC->PA at work->SF-12 score	.1225 (95% CI -.3753, .6064)	
EDUC->Sports PA->SF-12 score	.1241 (95% CI -.1037, .4590)	
EDUC->Leisure-time PA->SF-12 score	-.1331 (95% CI -.4802, .0833)	
EDUC->PA at work->Sports PA->SF-12 score	-.0258 (95% CI -.1400, .0281)	
EDUC->PA at work->Leisure-time PA->SF-12 score	-.0775 (95% CI -.1961, .0059)	
EDUC->Sports PA->Leisure-time PA->SF-12 score	.0736 (95% CI -.0096, .2150)	
EDUC->PA at work->Sports PA->Leisure-time PA->SF-12 score	-.0153 (95% CI -.0595, .0079)	

We show unstandardized regression coefficients (b) and bootstrap confidence intervals for the association. Education is codified into three levels: (1) < 3rd cycle of education, (2) secondary education, (3) higher education. PA is physical activity. PCS and MCS are Physical and Mental Component Summaries of SF-12. SF-12 score corresponds to the total score on the SF-12 scale. Proportion mediated (PM) or Proportion Suppressed (PS) represent the percentage of the total effect of Educational attainment on health-related quality of life by each pathway.

Table S8. Relationships between educational attainment and PA at work and total leisure-time PA (model 3) in health-related quality of life in the Madeiran population aged between 18 and 44 years old (N = 185).

Pathways coding education in 8 categories	Indirect effect	PM or PS
**Outcome PCS**		
Total (model)	.0089 (95% CI -.1714, .1705)	
EDUC->PA at work->PCS	.0136 (95% CI -.1597, .1525)	
EDUC->Total leisure-time PA->PCS	.0217 (95% CI -.0506, .1063)	
EDUC->PA at work->Total leisure-time PA->PCS	.0207 (95% CI -.0764, .0050)	
**Outcome MCS**		
Total (model)	-.0372 (95% CI -.3426, .2542)	
EDUC->PA at work->MCS	-.0315 (95% CI -.3300, .2434)	
EDUC->Total leisure-time PA->MCS	.0266 (95% CI -.0566, .1401)	
EDUC->PA at work->Total leisure-time PA->MCS	-.0323 (95% CI -.0960, .0050)	
**Outcome PCS**		
Total (model)	-.0284 (95% CI -.4931, .4231)	
EDUC->PA at work->SF-12 score	-.0179 (95% CI -.4327, .3829)	
EDUC->Total leisure-time PA->SF-12 score	.0483 (95% CI -.1162, .2206)	
EDUC->PA at work->Total leisure-time PA->SF-12 score	-.0587 (95% CI -.1724, .0132)	
**Pathways coding education in 3 categories**		
**Outcome PCS**	.0275 (95% CI -.2688, .2676)	
Total (model)	.0146 (95% CI -.2475, .2140)	
EDUC->PA at work->PCS	.0512 (95% CI -.0373, .1771)	
EDUC->Total leisure-time PA->PCS	-.0384 (95% CI -.1192, .0054)	
EDUC->PA at work->Total leisure-time PA->PCS		
**Outcome MCS**		
Total (model)	.0074 (95% CI -.4091, .4034)	
EDUC->PA at work->MCS	-.0094 (95% CI -.3958, .3727)	
EDUC->Total leisure-time PA->MCS	.0666 (95% CI -.0597, .2431)	
EDUC->PA at work->Total leisure-time PA->MCS	-.0499 (95% CI -.1549, .0050)	
**Outcome PCS**		
Total (model)	.0348 (95% CI -.5457, .6057)	
EDUC->PA at work->SF-12 score	.0053 (95% CI -.5516, .5322)	
EDUC->Total leisure-time PA->SF-12 score	.1179 (95% CI -.0910, .3779)	
EDUC->PA at work->Total leisure-time PA->SF-12 score	-.0883 (95% CI -.2496, .0036)	

We show unstandardized regression coefficients (b) and bootstrap confidence intervals for the association. Education codified into three levels corresponds to: (1) < 3rd cycle of education, (2) secondary education, (3) higher education. PA is physical activity. PCS and MCS are Physical and Mental Component Summaries of SF-12. SF-12 score corresponds to the total score on the SF-12 scale. Proportion mediated (PM) or Proportion Suppressed (PS) represent the percentage of the total effect of Educational attainment on health-related quality of life by each pathway.

Table S9. Relationships between educational attainment and PA at work and total leisure-time PA (model 3) in health-related quality of life in the Madeiran population ≥ 45 years old (N = 179).

Pathways coding education in 8 categories	Indirect effect	PM or PS
**Outcome PCS**		
Total (model)	.0073 (95% CI -.1190, .1322)	
EDUC->PA at work->PCS	.0167(95% CI -.0869, .1184)	
EDUC->Total leisure-time PA->PCS	.0158 (95% CI -.0770, .1106)	
EDUC->PA at work->Total leisure-time PA->PCS	-.0252 (95% CI -.0661, -.0002)	PS = -4.1%
**Outcome MCS**		
Total (model)	.0184 (95% CI -.1324, .1712)	
EDUC->PA at work->MCS	.0272 (95% CI -.1131, .1669)	
EDUC->Total leisure-time PA->MCS	.0148 (95% CI -.0661, .1194)	
EDUC->PA at work->Total leisure-time PA->MCS	-.0236 (95% CI -.0607, .0015)	
**Outcome PCS**		
Total (model)	.0257 (95% CI -.2354, .2832)	
EDUC->PA at work->SF-12 score	.0439 (95% CI -.1856, .2689)	
EDUC->Total leisure-time PA->SF-12 score	.0306 (95% CI -.1399, .1931)	
EDUC->PA at work->Total leisure-time PA->SF-12 score	-.0488 (95% CI -.1267, -.0006)	PS = -7.6%
**Pathways coding education in 3 categories**		
**Outcome PCS**	.0601 (95% CI -.2177, .3332)	
Total (model)	.0461(95% CI -.1856, .2736)	
EDUC->PA at work->PCS	.0688 (95% CI -.1015, .2431)	
EDUC->Total leisure-time PA->PCS	-.0547 (95% CI -.1289, -.0033)	PS = -4.5%
EDUC->PA at work->Total leisure-time PA->PCS		
**Outcome MCS**		
Total (model)	.0793 (95% CI -.2713, .4056)	
EDUC->PA at work->MCS	.0656 (95% CI -.2377, .3601)	
EDUC->Total leisure-time PA->MCS	.0667 (95% CI -.0925, .2955)	
EDUC->PA at work->Total leisure-time PA->MCS	-.0530 (95% CI -.1303, .0027)	
**Outcome PCS**		
Total (model)	.1394 (95% CI -.4550, .6543)	
EDUC->PA at work->SF-12 score	.1117 (95% CI -.3904, .5736)	
EDUC->Total leisure-time PA->SF-12 score	.1355 (95% CI -.2151, .5269)	
EDUC->PA at work->Total leisure-time PA->SF-12 score	-.1078 (95% CI -.2487, -.0003)	PS = -9.5%

We show unstandardized regression coefficients (b) and bootstrap confidence intervals for the association. Education codified into three levels corresponds to: (1) < 3rd cycle of education, (2) secondary education, (3) higher education. PA is physical activity. PCS and MCS are Physical and Mental Component Summaries of SF-12. SF-12 score corresponds to the total score on the SF-12 scale. Proportion mediated (PM) or Proportion Suppressed (PS) represent the percentage of the total effect of Educational attainment on health-related quality of life by each pathway.

Figures

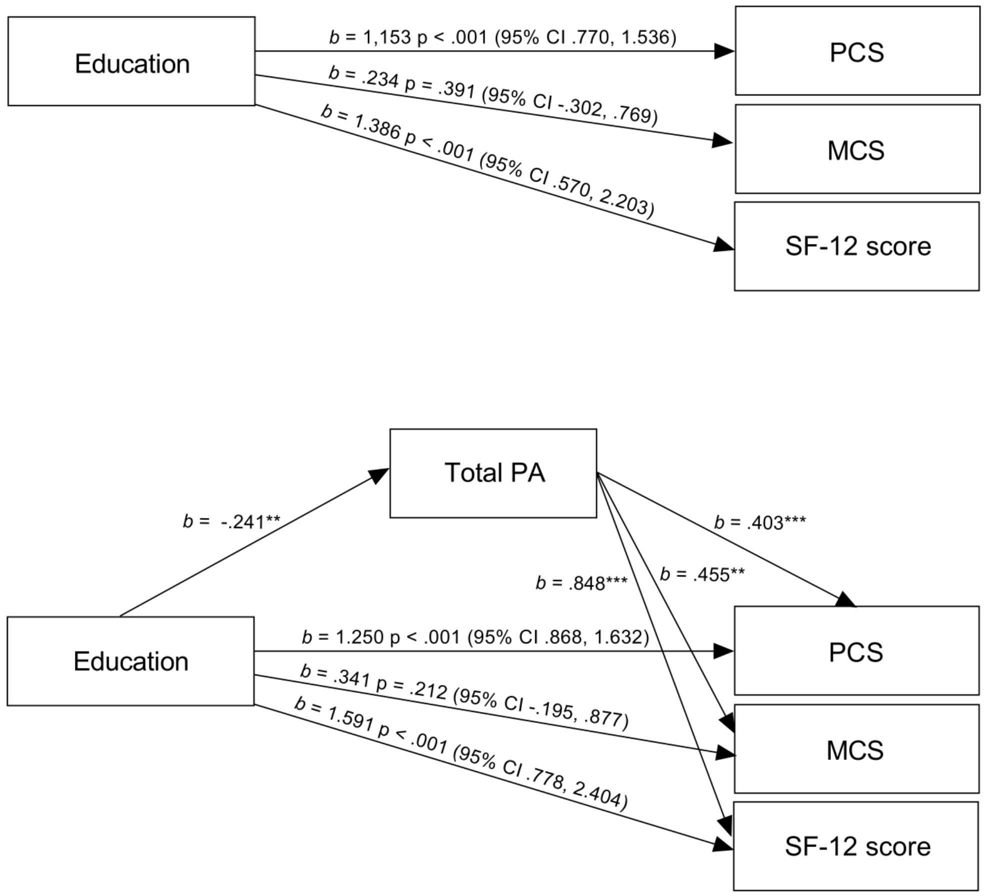

Figure S1. Relationships between educational attainment (coded in 3 categories) and total physical activity in health-related quality of life (N = 364). We show unstandardized regression coefficients (b) and bootstrap confidence intervals for the association. Education is codified into three levels: (1) < 3rd cycle of education, (2) secondary education, (3) higher education. PA is physical activity. PCS and MCS are Physical and Mental Component Summaries of SF-12. SF-12 score corresponds to the total score on the SF-12 scale. **P ≤ .01, ***P < .001.

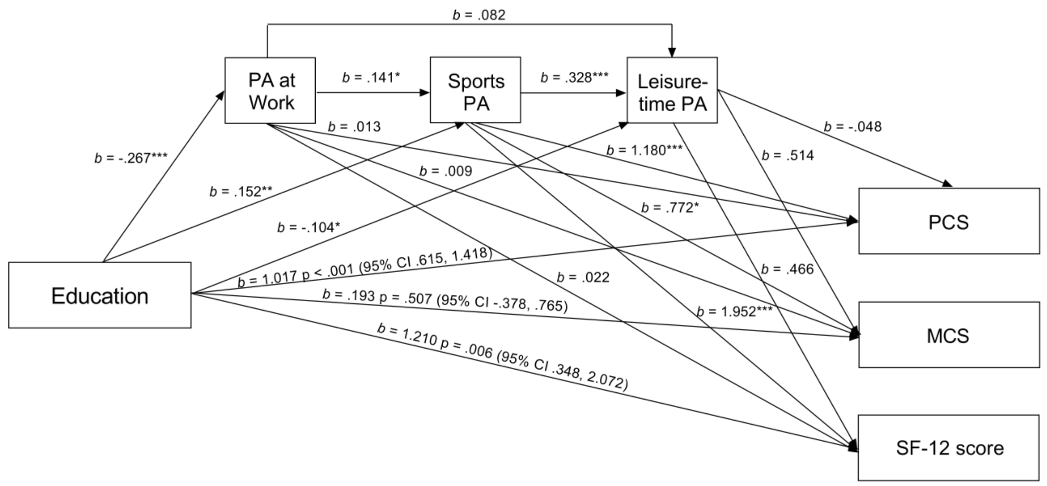

Figure S2. Relationships between educational attainment (coded in 3 categories) and work, sports and leisure physical activity in health-related quality of life (N = 364). We show unstandardized regression coefficients (b) and bootstrap confidence intervals for the association. Education is codified into three levels: (1) < 3rd cycle of education, (2) secondary education, (3) higher education. PA is physical activity. PCS and MCS are Physical and Mental Component Summaries of SF-12. SF-12 score corresponds to the total score on the SF-12 scale. *P < .05, **P ≤ .01, *** P < .001

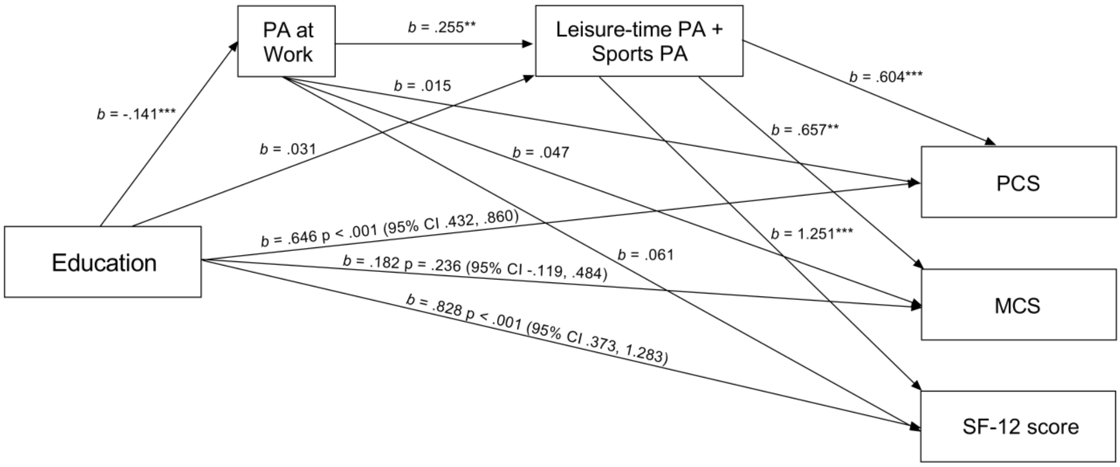

Figure S3. Relationships between educational attainment and work and total leisure-time physical activity in health-related quality of life (N = 364). We show unstandardized regression coefficients (b) and bootstrap confidence intervals for the association. Education is coded into eight levels: (1) no education; (2) 1st cycle; (3) 2nd cycle; (4) 3rd cycle; (5) secondary school level; (6) Bachelor Degree; (7) Master Degree; or (8) PhD. PA is physical activity. PCS and MCS are Physical and Mental Component Summaries of SF-12. SF-12 score corresponds to the total score on the SF-12 scale. **P ≤ .01, *** P < .001

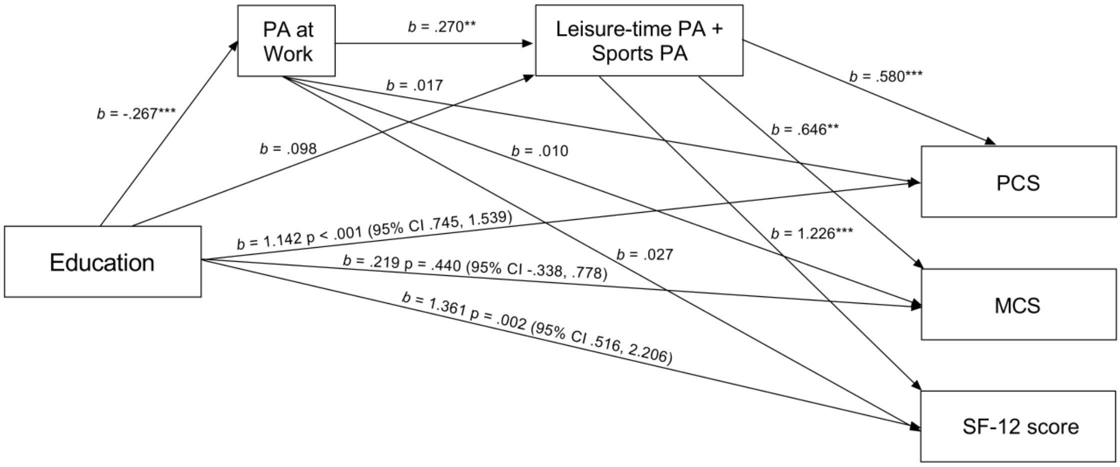

Figure S4. Relationships between educational attainment (coded in 3 categories) and work and total leisure-time physical activity in health-related quality of life (N = 364). We show unstandardized regression coefficients (b) and bootstrap confidence intervals for the association. Education is codified into three levels: (1) < 3rd cycle of education, (2) secondary education, (3) higher education. PA is physical activity. PCS and MCS are Physical and Mental Component Summaries of SF-12. SF-12 score corresponds to the total score on the SF-12 scale. **P ≤ .01, *** P < .001.
